# Supplementary material for: Factors influencing the delivery of telerehabilitation for stroke: A systematic review
Source: PLoS One. 2022 May 11;17(5):e0265828. doi: 10.1371/journal.pone.0265828 (PMC9094559; doi:10.1371/journal.pone.0265828)
Supplement: S2 File — (DOCX) [file pone.0265828.s002.docx]

| **Study ID** *(surname and year published)* |  | | |
| --- | --- | --- | --- |
| **Reference citation** |  | | |
| **Date form completed** |  | **Author extracting initials** |  |
| **Notes:** | | | |

## Methods

|  | **Descriptions as stated in the report/paper** | **Location in text or source** *(pg & ¶/fig/table/other)* |
| --- | --- | --- |
| Aim of study |  |  |
| Notes: | | |

## Participant Characteristics

|  | **Description** | **Location in text** |
| --- | --- | --- |
| Participant details | *Total no.:* |  |
|  | *Male/female:* |  |
|  | *Mean age +/- SD:* |  |
| Time post-stroke |  |  |
| Eligibility criteria related to: | *Intervention target (mobility, speech etc):* |  |
|  | *Cognitive deficits:* |  |
|  | *Communication difficulties:* |  |
|  | *Computer literacy access:* |  |
| Notes: | | |

## Intervention arms

|  | **Description** | **Location in text** |
| --- | --- | --- |
| Description of the intervention arm | *Aim/description:* |  |
|  | *System (mode of delivery, features, technical requirements) :* |  |
|  | *Provider:* |  |
|  | *Co-interventions:* |  |
| Dose / duration of the intervention: |  |  |
| No. in intervention group  *No. of dropouts (reasons)* |  |  |
| Description of the control arm |  |  |
| No. in control group  *No. of dropouts (reasons)* |  |  |
| Notes: | | |

## Outcomes

|  | **Description as stated in report/paper** | **Location in text** |
| --- | --- | --- |
| Primary clinical outcome | *Clinical outcome:* |  |
|  | *Outcome measure:* |  |
|  | *Timepoint measured:* |  |
|  | *Findings/results:* |  |
| Adherence | *Measurement:* |  |
|  | *Timepoint measured:* |  |
|  | *Findings/results:* |  |
| Other user experience outcomes | *Clinical outcome:* |  |
|  | *Outcome measure:* |  |
|  | *Timepoint measured:* |  |
|  | *Findings/results:* |  |
| Notes: | | |

## Practicalities of telerehabilitation intervention

|  | **Description as stated in report/paper** | **Location in text** |
| --- | --- | --- |
| Cost |  |  |
| Requirements | *Equipment (access/provided):* |  |
|  | *Training needs (participant / carer / clinician):* |  |
|  | *Clinical support needed:* |  |
|  | *Technical support needed:* |  |
|  | *Carer support needed:* |  |
|  | *Other:* |  |
| Uptake / attendance *(qualitative report not covered in drop-outs or adherence)* |  |  |
| Positive aspects/ benefits of intervention |  |  |
| Barriers/ Limitations of intervention (technical problems / adverse events) |  |  |
| Key conclusions of study authors / explanation of findings |  |  |
| References to other relevant studies |  |  |
| Notes: | | |
