# Supplementary material for: Factors influencing the delivery of telerehabilitation for stroke: A systematic review
Source: PLoS One. 2022 May 11;17(5):e0265828. doi: 10.1371/journal.pone.0265828 (PMC9094559; doi:10.1371/journal.pone.0265828)
Supplement: S3 File — (DOCX) [file pone.0265828.s003.docx]

Summary of telerehabilitation intervention characteristics

| **Study ID** | **Participant clinical characteristics** | **Mode of delivery and Access** | **Training and support** | **Use** | **Qualitative feedback** |
| --- | --- | --- | --- | --- | --- |
|  | | | | | |
|  | Treatment target  Time post-stroke/phase  Relevant exclusion criteria | Platform used / Features / Technical requirements  Access / Provided / Costs | Training for participant / carer / clinician  Clinician / Carer / Technical support | Parameters / Dose  Adherence /  Engagement /  Attrition | Technical problems / adverse events  Barriers / Facilitators / Challenges  Explanation of findings |
|  | 4a-I, 15a-I, 21-I | 5-vii, 5-viii | 5-x, 5-xi, 5x-ii | 5-ix, 6a-ii, 13b-i, 17a-i | 19-I, 19-ii |
| Asano 2019 | Function.  Included participants up to 4 weeks post-stroke.  Excluded participants with cognitive impairment.  Participants required to have independent sitting balance, mobile with assistance by one. | Rehabilitation programme delivered by telerehabilitation system with **remote monitoring and video-consultations**.  Hardware component – **iPad** with stand to record therapy, two limb sensors, a heart rate and blood pressure monitoring set.  Software - **MySQL relational database management system** to store data such as exercises selected, video and sensor data.  **Internet access** required.  Costs NR. | Participant and carer received training how to use system and perform TR exercises. Usually 1–3 1-hour sessions, including competency checklist, also baseline visit at home for set up. Patient guide within the application for additional help on its usage. Videos sent to participant if exercises are changed remotely.  Clinician - monitors and modifies exercise prescription remotely.  Other support NR. | Therapy 5 days per week, plus video call once per week, for 3 months.  IG completed 295 mins TR, accessed system on 22 days, completed 10 video calls.  Similar overall time spent on therapy in IG (2577 mins) and CG (2565 mins).  11/61 dropouts in IG; 15/63 dropouts in CG; reasons NR. | Adverse events NR.  TR led to improved accessibility to  the clinicians; this might have encouraged our participants to continue with other rehabilitation, contributing to a trend of increased time spent on centre-based rehabilitation by the IG. |
| Bishop 2014 | Quality of life and healthcare utilisation in stroke survivors and carers.  Included participants up to 6 months post-stroke on discharge from hospital.  Excluded participants with cognitive deficit.  Participants required to have a carer. | **Telephone contacts** with both stroke survivors and carers separately after hospital discharge to deliver intervention, plus provided with written information and resources.  Cost NR. | Participant training NR. Clinician training involved didactic instruction,  familiarisation with the manual, role playing, and group supervision.  Clinicians delivering intervention were a psychiatric resident, a family therapy graduate student, a stroke  rehabilitation nurse, and a master’s level family therapist.  Carer received intervention calls also. | Telephone calls were 15 minutes (mean), once weekly for 6 weeks, biweekly for 2 months, then monthly for 2 months. Total of 13 calls to each individual (26 calls per dyad).  Average contact per dyad was 4.7 hours.  NSD in dropouts in IG and CG.  8/49 dropouts; n=3 self-withdrew or failed to return calls. Group allocation unclear. | No adverse events.  “Telephone tag” impeded intervention delivery.  Participants appreciated the contacts and were generally easy to engage.  Initial calls were longer (30-40 minutes), later calls were shorter (5-10 minutes). Shorter calls were still effective.  The consistent and repeated format and the use of a grid for participants to place in front of them during calls structured and focused each contact. |
| Boter 2004 | Quality of life and satisfaction with care.  Stroke phase NR. Participants recruited prior to discharge home following stroke.  Eligibility related to communication and cognitive deficits NR. | Outreach care program on stroke prevention, stroke services and individualised support via 3 **telephone calls** and 1 home visit.  Costs NR. | Participant training NR. Nurses were comprehensively trained.  Nurses supported patients and carers according to needs (e.g. information, reassurance, advice to contact the general practitioner).  Other support NR. | 3 telephone calls + 1 home visit, within 5 months post-discharge.  231/263 (90%) of IG completed primary outcomes; n=1 IG and n=1 CG withdrew due to dissatisfaction with care. | NR. |
| Carey 2007 | Brain reorganization and recovery of hand function.  Included participants were veterans >12 months post-stroke.  Mean time post-stroke: IG1=42.5±24.3 months; IG2=35.6±26.1 months.  Eligibility related to communication and cognitive deficits NR.  Participants required ≥90^o^ passive and ≥10^o^ active extension-flexion at the index finger metacarpophalangeal joint of the paretic hand. | **Mobile phone and Webcam teleconferencing** plus finger and wrist training completed on a laptop with customised tracking software.  Therapist was unable to monitor training effort or performance records remotely. Records retrieved when participant returned the equipment.  Laptop (Dell Latitude 600), custom-made training software, cellular phone and a **Web camera** that operated over the **Internet connection** using the participants **telephone line.**  Equipment was issued to each participant.  Cost NR. | During orientation, the participant practiced setup once under therapist supervision, then took the equipment home.  Participants watched a video showing the training setup procedures.  The therapist had a pager and was available to answer specific questions if the subject called.  Carer and technical support NR. | The therapist contacted the participant about 5 times in total over the 10 training sessions using a cellular phone and Web camera.  2/12 dropouts in IG1; 0 dropouts in IG2. | NR. |
| Chen 2017 | Physical function for stroke survivor with hemiplegia and to reduce carer burden.  Included participants 14-90 days post-stroke  Mean time post-stroke: IG=24.96 ± 5.62 days; CG: 26.85 ± 4.68 days  Excluded participants with cognitive impairment. | TR supervised by **videoconference**.  TR system included a **network data system** for video conferencing, rehabilitation education and consultation, therapy and assessment, data collection, record, and storage, with sufficient bandwidth (>10 Mbps). Therapist end included high-quality **video-audio system**, remote control system, and electronic medical records system. Patient end included **video-audio system**, muscle electricity biofeedback instrument, and **physiological data collection system**.  Access and cost NR. | Prior to discharge therapists provided training on the exercises and use of system provided until the participants or the carers could manage it without the help of the therapists.  Clinician supervised intervention by videoconferencing and monitored log, vital signs and EMG signal.  Carers supervised and assisted with rehabilitation and maintaining therapy log to document participant’s daily training.  Therapists were available if the survivors and carers had problems. | Exercises for 1 hour + electromyography-triggered neuromuscular stimulation for 20 minutes, both twice per working day, for 12 weeks.  1/27 dropouts in IG (refused); 2/27 dropouts in CG. | Adverse events NR.  Reliance on carer support with intervention, but this did not increase carer burden. |
| Chumbler 2010 | Functional mobility.  Included participants were veterans up to 24 months post-stroke.  Median time post-stroke: IG=26 days; CG=74 days.  Excluded participants with cognitive impairment. | **Video-recorded televisits-** recorded by researcher and reviewed by therapist later, **in-home messaging device (IHMD)** for communication between the teletherapist and the participant, and **telephone call** **reviews** with the teletherapist to problem-solve barriers to exercise and to review and advance the exercise programmes.  Researcher used a **camcorder** to record both the home environment and the participant carrying out standardized measures of physical and functional performance. The **IHMD** (provided) was connected to a **standard telephone line** in the participant’s home. Participant’s own phone and phone line .  Cost NR. | Participant training NR. Researcher recording televisit trained to assess physical performance and instruct on exercises, assistive technology and/or adaptive techniques recommended by a licensed PT or OT (teletherapist).  Researcher video-recorded home visits; therapist communicated with the patient via telephone messaging to establish rapport and review the participant’s exercise regimen and assistive technology and explore solutions to barriers identified. Therapist also remotely monitored use of the IHMD weekly.  Carer and technical support NR. | **Daily use of an IHMD**, 3 home televisits, every 12-16 days for 5 weeks, and 5 telephone calls approximately every 14 days, with the first occurring 7 to 10 days after Televisit 1. The intervention lasted 3 months.  5 dropouts, mainly unable to contact or unwilling to do final outcome assessment. | No serious study-related adverse events occurred.  Challenges to the implementation of this intervention included variable infrastructure of technology in the home (e.g., availability of high-speed internet), information security when using the internet, and technological needs for accurate real-time recording.  To compensate for some of these issues, a combination of store & forward technology (ie, videotaping all encounters) in the event of technological issues affecting the accuracy with the live 2-way video.  A fourth televisit was requested by the teletherapist in the event of interval problems that could not be addressed via telephone. |
| Cramer 2019 | Upper limb movement.  Included participants were 4-36 weeks post-stroke with mild to severe arm motor deficits.  Mean time post-stroke: IG=132±65 days; CG=129±59 days.  Excluded participants with cognitive and communication deficits interfering with participation. | Upper limb exercises and functional training (including games and education) via an in-home **internet-connected computer** with **videoconference** review to discuss progress, issues, and revise treatment plans as needed.  TR system included:  **Internet-connected computer** with 23.8-inch monitor (Lenovo B50 with Intel Core i5-4460 processors and 8GM RAM with built-in webcam running Windows 7 Home Premium Edition), Verizon **wireless modem** (Jetpack MiFi 6620L), and 12 gaming input devices, but no keyboard, as no computer operation was required by patients. **System** **software** supported videoconferencing and organised the 70 minutes of therapy. The 12 input devices used for therapy were a **camera for videoconferencing**, a PlayStation Eye camera, motion game controller (PlayStation Move), joystick, small buttons (10), large buttons (4), toy gun holding a Wii remote with corresponding IR sensor bar, trackpad (Logitech), grip force cylinder, pinch force cube, rotating shuttle wheel (Powermate, Griffin), and three-axis gyroscope/ accelerometer/ magnetometer (Myo armband; no EMG features were employed). Device usage was selectively assigned by therapists based on clinical judgment.  Kit all provided where required.  Cost NR. | Participants were trained to use the TR system at the baseline visit.  Clinician conducted videoconference review.  Technical support: Each TR system also ran software (Teamviewer) that enabled study team members to remotely access the home-based TR computer, which could be used to troubleshoot certain problems.  Carer support NR. | Sessions of 70 minutes + 10-minute break, 36 sessions (18 supervised and 18 un-supervised) during a 6- to 8-week period.  Adherence was 98.3% in IG, 93.3% in CG. NSD between groups.  Participants in both groups were adherent (completed >40/70 minutes therapy) with median 18 (interquartile range, 18-18)  supervised and unsupervised sessions .  n=3 dropouts in IG, all before first therapy session; n=7 in CG. | Adverse events: 6 IG participants experienced arm and shoulder pain and 5 CG participants experienced fatigue and arm and shoulder pain. There were no study-related serious adverse events.  Technical assistance was required included program navigation and wireless connectivity issues. This decreased in frequency over time. Months 1 – 2 one call per week for assistance, months 3-4 one call every 2 weeks.  High levels of satisfaction in both IG and CG. CG reported slightly higher satisfaction at the end of therapy (mean [SD], 55.2 [7.7]vs 58.5 [8.0];P= .02). Higher satisfaction in the CG group suggest areas for improving TR therapy, possibly by increasing time spent interacting with a therapist. |
| Deng 2012 | Ankle dorsiflexion during the swing phase of gait in people with stroke.  Included participants were >5 months post-stroke.  Median time post-stroke: IG1 = 66 months; IG2 = 16.5 months.  Participants required at least 10 degrees of active dorsiflexion/plantar flexion at the paretic ankle, ability to under-stand the tasks, and ability to ambulate 30 m.  Eligibility related to communication and cognitive deficits NR. | TR exercise via a laptop using customised tracking software and regular **videoconferencing** between the therapist and the participant. The computer automatically emailed daily records to the laboratory computer to allow **remote monitoring** of performance and compliance.  **Laptop computer** (Dell VOSTRO1,000) with the customized training software. A **remote desktop application** **(LogMeIn)** and a **video conferencing application (Skype)** were used for the communication, along with **webcam** (Logitech). The **Internet connection** was made using a cellular modem (Verizon) communicating with a cellular phone network (Verizon).  Equipment provided where required.  Cost NR. | The participant and a family member were instructed to set up the computer/camera and apply the customised electrogoniometer with an attached potentiometer. Several training trials (with participant and carer) were observed to ensure competency.  Clinician remotely monitored performance and conducts TR visits.  Technical support NR. | 60 training blocks per day, with 3 trials per block, for 20 days for a total of 3,600trials. TR contact with therapist was twice per week.  IG1 n=10 dropouts, n=2 due to fatigue; IG2 n=9 dropouts, n=1 due to fatigue. | Apart from report of fatigue in the dropouts, there were no other adverse events.  Teleconferencing connection was generally successful in allowing 2-way  video and audio communication. For 4 participants the wireless signal was inadequate in their homes. In these cases, telephones were used for audio communication and data were retrieved from the computer’s hard drive after they finished the training.  Participant feedback was very  favourable for TR. The most frequently reported difficulty was donning the electro-goniometer (non-TR component); some participants required partial assistance from the primary carer, particularly at the beginning and less so toward the end. |
| Forducey 2012 | Physical and psychosocial function.  Included participants were up to 6 months post-stroke and had moderate deficits in self-care, functional mobility and transfers.  Excluded participants with cognitive and communication deficits. | Technology facilitated direct **video calls** (for education, assessment, treatment) with PT and OT.  Desktop videophone using standard telephone lines (H.324 standard compliant device). The system included external audio-video devices, cameras, monitors, projection devices and television displays. Maximum transfer rate = 33.6 kbps with video streaming at 20-24 frames per second, which facilitated observation of the person with stroke's physical presentation and movement. patterns.  The videophone equipment expenses were low- exact cost NR. | Participant training NR. PT and OT with ≥4 years rehabilitation experience and ≥6 months in the home health setting.  Carer required to set up videophone device.  Technical support NR. | Two sessions per week (1 OT and 1 PT), over approximately 6 weeks.  IG received 6.0 PT and 6.75 OT treatments, CG received 10.6 PT and 7.4 OT treatments.  n=2 dropouts overall, group allocation and reasons NR. | Adverse events NR.  The PT and OT could adjust the frames-per-second to observe a clearer picture, although motion quality was somewhat reduced.  The technology was “user friendly”. |
| Grau-Pellicer 2019 | Physical activity.  Mean time post-stroke IG=18.92±27.60 months; CG=20.85±59.74 months.  Excluded participants with cognitive impairment. | **Digital platform** including **bidirectional feedback** with the researchers, group rehabilitation program (aerobic, task-oriented, balance and stretching exercises), progressive daily app- and pedometer-driven walking program, **WhatsApp group** for motivation, feedback and collective identity in the group.  The digital platform included 2 apps **Fitlab® Training** and **Fitlab®**(www.HealthSportlab.com, Barcelona). Features included GPS and accelerometer to monitor walking distance and speed and supervise adherence to PA; questionnaires to assess mood, effort, recovery, wellness and fatigue; bidirectional feedback to visualise results and exchange messages with the researchers.  Access and costs NR. | Training in use could be provided at the in-person sessions if required.  Group rehab is PT-led. Participants could exchange messages with the researchers via app and WhatsApp.  Carer and technical support NR. | 8-week programme but the app and WhatsApp could be used as and when required.  50% compliance rate with app. | No adverse events.  Frequent support and guidance of researchers and carers was required.  Main inconveniences with the app were due to technical issues (internet connection, not proper device or too complicated procedures for a regular use).  Recommendation to develop stroke-friendly, easy use procedures to improve adherence.  Participants described difficulties using smartphones and the Apps (challenging to use). The most accepted device was the pedometer, due to the ease of use.  Participants positively valued the combination of the program at the rehabilitation unit with the digital platform based on the app and the pedometer. Participants valued the in-person component due to supervision using the app and encouragement to be active (group exercise program and guided progressive ambulation program at home). The WhatsApp group encouraged adherence to the program and the use of the app, due to feedback from the professionals and the researcher. This increased self-confidence. |
| Huijgen 2008 | Upper limb function.  Mean time post-stroke: IG=3±2 years; CG=1.8±0.8 years.  Excluded participants with communication problems.  Participants required to have Internet connection or telephone line and reachable Internet provider. | Upper limb rehabilitation using the Home Care Activity Desk (HCAD) system, which video-recorded therapy for **remote monitoring** by therapist, plus **videoconference review**.  The unit has 2 **webcams** for videoconferencing and recording.  The **HCAD system** comprised a hospital-based server and  the portable unit which was installed at the patient’s home. Kit included a key, light bulb, book, jar, writing, checkers and keyboard.  Cost NR. | Participants received 4 training sessions with the system in the hospital.  Therapists installed the system at home.  Other support NR. | 30-minute training sessions, at least once a day, 5 days a week, for one month.  Videoconference once per week.  IG 7/55 dropouts: n= 1 no interest, n=1 1 individual training impossible.  CG 4/26 dropouts.  IG used the  system for average 19 (range 7-38)  days for 30 minutes per day.  Average treatment time IG (9.5 hours per month) was similar to CG (9 hours usual care per month). | Adverse events NR.  Participants and therapists were less satisfied with aesthetic aspect of the system and the difficulty of the tasks.  A key advantage is that IG could train more intensively than CG.  TR reduced time required by therapists compared to usual care.  CG had on average 3 in-person sessions with therapist versus IG had 1 videoconference with the therapist.  Reduced time investment and travel time needed, suggests that TR might be more efficient than usual care. |
| Joubert 2020 | Secondary stroke prevention.  Included participants <3 months post-stroke.  Excluded participants with severe cognitive impairment.  Participants required to have a carer. | **Telephone** follow-up between care-coordinator and stroke survivor, carer and family for education, advice and support, with frequency based on stroke risk factors.  Cost NR. | Training NR.  Carer required to participate in telephone follow-up and the  return of documentation to the coordinator.  Other support NR. | Telephone call on a weekly, monthly or three-monthly basis depending on stroke stratification of risk profile (mild, moderate and severe).  IG 77/112 and CG 81/137 completed final visit. Dropout reasons included declining interest, transport difficulties, irritation with telephone follow-up and lack of understanding of the importance of risk factor control. | NR. |
| Kirkness 2017 | Post-stroke depression.  Included participants up to 4 months post-stroke with clinical depression.  Eligibility related to communication and cognitive deficits NR. | Psychosocial behavioural intervention delivered by **telephone** sessions.  Cost NR. | Participants had one in-person orientation session, where they received the participant manuals, discussed goals and expectations of each session, and learned how to fill out the homework sections, either in their home or at study offices.  TR delivered by psychosocial nurse practitioner therapist  A family member or informal carer could participate and provide data with the participant’s agreement. | Telephone calls of 10-80 minutes, 6 calls, over 6 weeks.  Telephone calls were shorted than in-person contacts (average 26 minutes versus 38 minutes).  Similar dropouts and reasons in all groups. IG 3/37 dropouts, n=2 refused; CG1 3/35 dropouts, n=1 refused; CG2 3/38, n=1 refused and n=1 no response. | No harms attributable to the study were identified.  At the end of each session, the participant provided an evaluation of the session - NR.  Providing the intervention by telephone was as effective as conducting it in-person.  Greater accessibility by telephone. |
| Li 2020 | Functional assessment.  Participants recruited during inpatient hospitalisation.  Mean time post-stroke: 90.7±13.8 days.  Participants with cognitive or communication deficits were excluded.  Participants required to have a mobile phone. | Post-discharge assessment of functional tasks via **videoconference** (IG) or **telephone call** (CG).  Contact using the **WeChat app** on **mobile phone**. Not provided.  Costs NR. | Participants trained on use of videoconference function of the WeChat app prior to discharge.  Video/telephone calls were by researcher and they received training on how to use WeChat and video/telephone calls  Carer and technical support NR. | 2 video calls: first within 2 weeks post-discharge, second at 3 months post-discharge.  Duration of calls was not measured.  Video group = 11/60 dropouts due to not answering call or refusing to complete assessment); Telephone group = 12/60 dropouts due to not answering call. | Adverse events NR.  Patients reported higher satisfaction and confidence with videoconference assessment compared with telephone.  N=21 declined to participate in study due to concerns about use of mobile health technology. |
| Lin 2014 | Balance.  Included participants were >6 months post-stroke recruited from long-term care facilities.  Excluded participants with severe cognitive or communication deficits. | Balance training delivered via multi-user telerehabilitation system supervised by **videoconferencing** with vital signs monitoring.  System - **Data centre**.  Therapist end: **Notebook computer, internet access, Logitech webcam, videoconference sound recorder and switch hub**.  Participant end: **personal computer, screen for video communication, touch screen for interactive games, Logitech webcam**.  Pulse oximeter and blood pressure monitor sensors measured online using the **ZigBee wireless.**  **Equipment** and **internet access** required for both clinician and participant.  Costs NR. | Operational technique training of the user, family members and the therapist was required.  Therapist delivered balance training and monitored performance and vital signs.  Standby supervision or assistance is provided by volunteers or non-medical personnel.  Technical support NR. | 50 min sessions, 3 a week, for 4 weeks  1/12 dropout in the IG. | Adverse events NR.  Good level of participant satisfaction, including perceived usefulness, ease of use and attitudes towards use.  Two patients could be reviewed remotely by the therapist at the same time.  Cost of system may not be feasible.  Vital signs and videos of each patient could be stored for asynchronous monitoring. |
| Llorens 2015 | Balance for stroke survivors with hemiparesis.  Included participants were >6 months post-stroke with residual hemiparesis.  Mean time post-stroke: 325±55.3 days.  Excluded participants with severe cognitive or communication deficits. | Balance training via **remotely monitored** **exergame** plus **telephone contact**.  System used **television, conventional computer**, and a **Microsoft Kinect** (motion-sensing input device).  Participants used their own television and laptop was provided.  Cost analysis:  Overall expenses per participant in IG = $835.61. This was $654.72 less than in-person.  Equipment costs (laptop, Kinect, and Internet access) - $800 | Training NR.  Physical therapist explained the procedure, provided technical support and set initial difficulty level and adjusted if required.  Progress was checked remotely once weekly.  Weekly interview with therapist to troubleshoot technical problems.  Carer support NR. | 45 minutes sessions, 3 a week. 20 sessions in total.  Adherence not reported.  0 dropouts in IG. | Adverse events NR.  Cost benefit mainly due to reduced transportation services.  High levels of usability and motivation reported. |
| Maresca 2019 | Aphasia.  Time post-stroke NR.  Excluded participants with severe cognitive impairment . | **Tablet-based** rehabilitation training for aphasia using virtual reality rehabilitation system (VRRS-Tablet) with **videoconference** review.  The **VRRS-Tablet** contains about 30 different exercises, with over 1000 customizable and editable levels, divided into cognitive and linguistic modules, with exercises on attention, memory, perception, executive functions, and speech/language abilities.  The VRRS-Tablet is equipped with patient and therapist mode: the former allows performing the exercises and monitoring the data produced, while the latter allows monitoring the patient's progress through the platform.  VRRS-Tablet provided to exercise at home. It is controlled remotely from a workstation, called Cockpit.  Cost NR. | Participants and carers were trained to use the instrument and the software.  Neuropsychologist remotely monitored progress and conducted videoconference.  Carer support required.  Technical support NR. | 50 minutes sessions, 5 days a week for 24 weeks (12 in hospital, 12 at home). Videoconference twice a week during at-home phase.  Dropouts NR. | Adverse events NR.  The patients were dissatisfied by the improvements obtained, as they expected a nearly complete recovery of language abilities after the training. Considering that the recovery was partial, the perception of their quality of life has been negatively influenced. |
| Mayo 2008 | HRQoL and reducing unplanned health service use.  Included participants at discharge from the acute-care hospital post-stroke  Time post-stroke NR, but mean length of stay was 12.5±13.8 days.  Included participants with mild cognitive impairment. | **Telephone** intervention. Geriatric nurse case managers completed post-discharge assessment, planning, facilitation and advocacy via home visits and telephone contacts. They contacted participants’ physicians or health centres.  A 24-hour contact number was available.  Costs NR. | Participant, family, carer, physician training NR. Nurses were trained in assessment and documentation.  Nurse interactions included surveillance (91%), information exchange (80%), medication  management (70%), health system guidance (41%), active  listening (34%), family support (31%), teaching (23%), risk identification (19%).  Nurse communicated with family/carer but support NR. | On average each  participant received 7.8 telephone contacts lasting 5–20 min, during a 6-week intervention.  Interactions seemed to be on a needs basis, rather than scheduled.  Similar number of dropouts in IG (n=15) and CG (n=18). | Adverse events NR.  Passive intervention (health surveillance, information, education and psycho-social support) used in isolation without other rehab/ services may not be sufficiently potent to alter perceived health status. |
| Meltzer 2018 | Speech and language impairments - aphasia and cognitive-linguistic communication disorder (CLCD).  Included participants were >6 months post-stroke with aphasia or CLCD.  Excluded participants with cognitive deficits.  Included participants with ability to operate iPad. | **Remotely monitored computer-based exercises** plus remote therapy conducted via **teleconferencing** equipment and software. Oral and written feedback and phone calls for support with compliance.  Exercises using commercial **software TalkPath** on **iPad** or **computer system**. All participants loaned an iPad with TalkPath software for the duration of the study.  **Teleconferencing equipment** and **software (WebEx or VSee)** required but not provided. If unavailable, attended a telehealth centre for videoconferencing, or attended therapy site but without contact with therapist.  Costs NR. | Instruction on using TalkPath system during initial in-person meeting (2 hours).  Speech and language therapist monitored exercises remotely and provided feedback, delivered videoconference therapy and telephone calls.  Carer was communication partner involved in all sessions.  Technical support NR. | 1 hour per week TR with therapist, for 10 weeks + individualised computerised therapy plan.  Sub-group of participants completed homework on Talk Path which logged use - Among these 19 participants, there was a slight tendency for CG to do more hours of homework (IG: 21.1 h; CG: 28.9 h), but this was NSD. | Adverse events NR.  All participants reported satisfactory audio–video quality.  No participants reported any trouble using the software.  Limited availability of the appropriate technology in clients’ homes. Some participants had to travel a short distance to receive the TR treatment, which may have reduced the quality of their experience compared to those who could participate from home. |
| Ora 2020 | Speech and language.  Included participants with aphasia.  Time post-stroke  IG n=16 ⩽3 months, n=5 3–12 months, n=11 >12 months.  CG n=12 ⩽3 months, n=4 3–12 months, n=14 >12 months.  Excluded participants unable to perform 5 hours per week of speech-language therapy due to medical or cognitive reasons. | Augmented language training via **videoconference**.  Videoconference via Internet to a study **laptop** in the participant’s home or in the rehabilitation ward where the participant was admitted. The **videoconference software Cisco**  **Jabber/Acano** from the “Norwegian Health Net” was installed in the study laptops given to the participants and in videoconference equipment at  the hospital. **LogMeIn software** was used to remotely control the participant’s computer. To ensure adequate confidentiality and meet data safety requirements, the videoconference was provided through encrypted software. The technical setup further included an  **external speaker** to improve sound quality and a **wide-angle web camera** to enable review of body  language and/or gestures. Used **Internet connection** available in the local setting (mobile, WiFi or broadband)  Costs NR. | Participants were given  training in the use of the computer software usually  lasting for 30–60minutes.  Clinicians were trained in how to use the therapy material within the TR context and in usage of equipment and software was provided through piloting of inpatients  (approximately 10hours).  TR delivered by a speech-language pathologist.  Other support NR. | 60minutes of video-conference therapy via a day, five days a week, for 4 weeks.  In some cases, more prolonged therapy time (70–120minutes) was delivered over fewer days per week, to adjust to the  participant’s timetable and other planned activities, and where participants were able to withstand long sessions.  Minimum dose ⩾16 sessions, with intervention period up to 32days to account for any expected logistic or technical challenges, as well as medical complications or co-morbidities.  0 dropouts immediately post-intervention. | No treatment-related adverse events, serious harms, or drop-outs directly related to TR.  Data from the usual care log showed that the CG on average received more hours of usual care than the IG. This may have been due to IG not receiving the normal amount of usual care due to an already demanding rehabilitation schedule, or that enrolment in the trial increased access to usual care in the CG (one participant in the CG seemed to facilitate more hours of usual care therapy). |
| Piron 2008 | Upper limb motor function.  Primary outcome was satisfaction.  Included participants with mild - intermediate arm motor impairment.  Mean time post-stroke: 13±2 months.  Eligibility related to communication and cognitive deficits NR. | **Virtual reality motor tasks via 3D motion tracking system** controlled remotely by therapist with **videoconference** monitoring.  **3D motion tracking system** (Polhemus 3Space Fastrak) recorded participant arm movements and gave visual feedback. Interaction with therapist via **two PCs**, one in participant’s home and the second at the hospital. The **data connection** used a TCP/IP protocol via an ISDN connection at a data rate of 128 kbit/s. The **telerehabilitation software** also managed the participant’s PC by remote control. A high-quality **videoconference system** for monitoring of the  rehabilitation sessions using three ISDN lines at a data rate of 384 kbit/s. Three **ISDN lines** installed in each participant’s home for the videoconference plus another line for the rehabilitative data transfer.  Costs NR. | Participants and carers were briefly trained to operate  the system, even though the equipment was entirely controlled from the remote hospital workstation.  Other support NR.  support | 1 hour of rehabilitation daily for one month.  All participants completed the assigned program. | Adverse events NR.  IG were able to engage in therapy at home via a user-friendly system.  Similar satisfaction in IG and CG.  Participant–physiotherapist interaction may have been enhanced by the videoconferencing system because it was an interactive one  and not a store-and-forward interaction. |
| Piron 2009 | Upper limb motor function.  Included participants 7-32 months post-stroke with mild - intermediate arm motor impairment.  Mean time post-stroke 13.3±5.5 months.  Excluded participants with severe cognitive or communication deficits. | **Virtual reality motor tasks via 3D motion tracking** system with **videoconferencing** observation and feedback by clinician.  Telerehabilitation system (**VRRS.net**) generated a virtual environment for 5 virtual upper limb tasks with real-time feedback on target and own performance. Therapist could view virtual task and performance remotely and provide additional feedback.  **Personal computer (PC)-based workstations (for patient and clinician)**.  Broadband **internet access**.  Access and cost NR. | Participants were trained to use the system and to execute the requested motor tasks adequately.  Physiotherapist observation and feedback via videoconference. Complete assistance by the therapist, who could remotely control all the commands.  Carer support NR. | 1 hour per day, 5 days a week, for one month. | Adverse events NR.  A reduction in broadband quality was reported at times, with a slowing of the data flow and blurring of the images.  Occasionally there was an unexpected interruption in the connection between the 2 workstations.  All patients completed the study and they did not experience problems in handling the VRRS.net® system.  VR system was able to provide components that have been demonstrated to be useful in motor learning: knowledge of performance, knowledge of results, trial and error, learning by imitation. |
| Rochette 2013 | Reducing unplanned use of the healthcare system and HRQoL.  Included participants within one month post-stroke (first mild stroke).  Excluded participants with moderate or severe cognitive deficits. | Multimodal (**telephone, Internet**,  and paper) support intervention.  Telephone calls on issues, family functioning and risk factors. Referral to other services or directed to physician when required.  **Telephone** and **internet access** required – unclear if provided.  Costs NR. | Training NR.  Intervention delivered by trained health care professional / rehabilitation specialist.  Other support NR. | Call frequency was once weekly for months 1&2, biweekly month 3, and monthly for months 4-6.  Mean length of calls was 14.1±9.5 minutes, with an additional 10.3±5.4  minutes of indirect time.  Additional mail/ email/ online support on stroke management was provided as required.  78.4% of the participants were reached after a minimum of 3 attempts to telephone. | Adverse events NR.  Qualitative feedback recorded has the following themes: (1) appreciation of support; (2) intervention helped in problem solving; (3) intervention increased insight.  Giving telephone number to participants/family (CG) is ineffective, as they will not use (6/94 used control group contact details). |
| Rodgers 2019 | Performance of ADLs.  Participants recruited post early supported discharge.  Median (IQR) time post-stroke IG= 73 (48-111.5) days post-stroke.  Additional measures to include participants with cognitive or communication difficulties. | Extended stroke rehabilitation service consisted of rehabilitation reviews (for identifying needs, goal setting and action planning) intended to be conducted by **telephone**.  Home visits were conducted (21%) where reviews could not be completed via telephone. Participants received appointment card with checklist of general rehabilitation issues and a summary of review with recommendations sent by post.  68% chance that TR is cost saving. Intervention cost £437/$632. The mean cost of resource utilization was lower in the intervention group: −£311 (−$450 [95% CI, −£3292 to £2787; −$4764 to $4033). | Training NR.  PT (56%), OT (28%), nurse (8%), SALT (5%), stroke coordinator (1%) or dietitian (1%) member of the early supported discharge team delivered the reviews.  Carer may be involved in reviews. | Five reviews were conducted at 1, 3, 6, 12 and 18 months post-discharge.  2 hours was allowed for each review including documentation and follow-up. | Adverse events NR.  Patients in the intervention group appeared to be more satisfied with some aspects of their care.  Telephone reviews were selected because they were more affordable and less disruptive to a participant’s daily routine than a clinic visit.  Video or more sophisticated technology were not used as many participants would have not had access or experience in using them. |
| Saal 2015 | Physical functioning.  Participants were recruited at discharge post-stroke. Time post-stroke NR.  Cognitive and communication status were not considered exclusion criteria. | Stroke support service delivered via **telephone** and home visit, educational sessions (in-person), written information and direction to internet for further information.  Access and cost NR. | Training NR.  Nurse and physiotherapist assessed participants and delivered tailored intervention to participant and their next of kin.  Other support NR. | Median of 12 contacts (61% via telephone, 31% in-person, rest via email, post or bi-monthly educational sessions), over 12 months.  57/322 participants consented (during admission) discontinued before randomisation (4 weeks post-discharge). Of those randomised 11/130 IG versus 24/135 CG were lost to follow-up. | NR. |
| Smith 2012 | Depression in stroke survivors and their carers.  Included dyad where female carer provided care at home to male stroke survivor, and either experienced at least mild depression.  Excluded participants with cognitive impairment. Stroke survivor could mail questionnaires if they had communication difficulties. | Web-based psychoeducational support for carers, including weekly videos, **email support**, signposts, encouragement by professional guide; Educational videos; **online chat** sessions with 4-5 carers and professional guide; **email and message board**; online library resource with information and links to other resources.  **Computer hardware,** including **USB headsets and microphones** for **Adobe Connect online chats,** and **Internet access**. This was provided if required.  Costs NR. | Online and hard copy tutorials were developed to train the website users and made available to all carers.  Nurse is professional guide.  Intervention is primarily aimed at carers.  Helpline provided for technical problems. Additionally, CGs were phoned halfway through intervention to check about technical difficulties. | 1 video per week, 2 online chats per week, for 11 weeks.  5/19 dropouts in IG. | Adverse events NR.  Carers perceived the intervention as credible and were willing to devote considerable effort to it. |
| Svaerke 2019 | Visuospatial neglect or homonymous hemianopia.  Included participants in the sub-acute stroke phase, 3-42 days post-stroke, with visuospatial neglect or homonymous hemianopia.  Mean time post-stroke: 19±13.1 days.  Cognitive screening but deficits not excluded if they were able to provide consent and carry out intervention. | Computer-based cognitive rehabilitation + **telephone** contact to review progress and support..  Scientific brain-training PRO system with 5 exercises that were progressed (9 levels) based on performance.  Access and costs NR. | 1:1 instruction on program use at the beginning of the intervention period.  Technical and carer support NR  Unclear who delivers intervention. | 30-45 minutes every second day for 3 weeks plus weekly telephone call.  3/18 did not complete the intervention (1 did not start intervention, 2 did not wish to continue with intervention) – Group allocation NR.  IG completed 515±258 hours intervention, CG completed 446±191 hours intervention. | Adverse events NR.  5 participants required additional training due to problems with 1 or more exercises. |
| Torrisi 2019 | Cognitive function.  Included participants in the subacute phase (3–6 months post-stroke).  Eligibility related to communication and cognitive deficits NR. | **Tablet-based** cognitive rehabilitation using virtual reality rehabilitation system (VRRS) with real-time interaction via **videoconference**.  **VRRS** controlled remotely from a workstation, called **TeleCockpit**.  Cost NR. | Training NR.  Psychologist monitored the progress of rehabilitation.  Other support NR. | Phase 1 (in hospital): 50-minute sessions, 5 days a week for 12 weeks.  Phase 2 (at home): 50-minute sessions, 3 times a week, plus remote monitoring via video conference twice a week.  Dropouts NR. | Adverse events NR.  Positive: intervention encourages a more active participation and allows longer training sessions, enhances patients’ motivation and enjoyment. |
| Wan 2016 | Health behaviour.  Included participants hospitalised within 1 month post-stroke.  Excluded participants with severe cognitive or communication deficit. | **Telephone** follow-up program for goal setting, action planning, education, problem solving and encouragement plus usual stroke education and care.  Access and costs NR. | Participant training NR. Stroke nurses received intensive training in intervention delivery.  Telephone calls with stroke nurse.  Other support NR. | 15-20 mins telephone calls at week 1 and months 1 and 3 post-discharge (3 calls in total).  Similar dropouts and reasons in IG (n=6) and CG (n=5). | Adverse events NR.  Most patients did not set measurable behavioural goals or develop action plans, especially in the recovery phase from 3-6 months after discharge.  Feasible, convenient, and low-cost post-stroke intervention. |
| Wang 2019 | Secondary prevention - health behaviours, medication adherence, blood pressure, disability, and stroke recurrence.  Participants recruited during hospital admission (n=114 were <1year post-stroke; n=8 were 1-2 years post-stroke; n=29 were >2 years post-stroke)  Excluded participants with communication or cognitive difficulties.  Included participants with a mobile phone and the ability to access the phone and text functions. | Text message reminder system plus 3 **telephone call** follow-ups.  Participants were required to have a **mobile phone**.  Cost NR. | Prior to discharge, participants received intervention-related health education. Participants received a calendar handbook based on the Health Belief Model. Not TR-specific training  Nurse-led telephone call.  Technical and carer support NR. | Months 1-3: once weekly text message and monthly telephone call; months 3-6: weekly text message plus one telephone call at 6 months.  7/87 IG participants withdrew from the study. | Adverse events NR.  Follow-up telephone calls gave patients a chance to discuss their present situation and any problems and reinforced health behaviour change. |
| Withiel 2019 | Memory functioning.  Included participants >3 months post-stroke.  Mean time post-stroke 41.7±44.8 days  Excluded participants with severe cognitive or communication deficits.  Excluded participants with inadequate computer proficiency. | **Computerised cognitive training monitored remotely** with **telephone** contact for compliance.  **Lumosity** computer programme accessible online. Games were adaptive with complexity based on performance.  Access and costs NR. | Training and support NR.  Unclear who delivered the intervention. | 30 minutes per day, 5 days per week, for 6 weeks.  27% dropouts; participants completed a mean of 25/30 training sessions completed (83%; SD=16.01; range 3–43 sessions). | NR. |
| TR – telerehabilitation.  NR – not reported. | | PT- physiotherapist  OT – occupational therapist  SALT – speech and language therapist | | ADL – activity of daily living. | |
