# Supplementary material for: Factors influencing the delivery of telerehabilitation for stroke: A systematic review
Source: PLoS One. 2022 May 11;17(5):e0265828. doi: 10.1371/journal.pone.0265828 (PMC9094559; doi:10.1371/journal.pone.0265828)
Supplement: S4 File — (DOCX) [file pone.0265828.s004.docx]

| **Study ID** | **Participant clinical characteristics**  Treatment target  Time post-stroke/phase  Relevant exclusion criteria | **Mode of delivery and access**  Platform used / Features / Technical requirements  Access / Provided / Costs | **Training and support**  Training for participant / carer / clinician  Clinician / Carer / Technical support | **Use**  Parameters / Dose |
| --- | --- | --- | --- | --- |
| Allegue  2020 | Upper limb motor function.  Includes participants in the chronic stroke phase.  Excludes participants with severe cognitive or communication deficit.  Participants require sitting balance. | Jintronix exergame + videoconference review with therapist.  Participants provided with **computer**, Kinect camera, Reacts software, Jintronix software and **internet** **access via USB internet key**.  Costs NR. | Participants receive 30-minute training session with technician.  OT or PT remotely monitor and videoconference with participants.  Technician transports and installs system and provides initial training.  Carer support NR. | Jintronix for at least 30 minutes, 5 days a week, for 8 weeks (total= 20 hours).  Videoconference sessions 3 times a week for 2 weeks, twice a week for 2 weeks, and once a week for remaining 4 weeks. |
| Blanton 2019 | Physical and psychosocial health (both carer and stroke survivor).  Includes participants 1-24 months post-stroke with minimal to moderate upper limb deficit.  Excludes participants with severe cognitive deficit. | Online interactive educational modules (text and video) for carers to support home-based CIMT with **self-reflection questions**. Responses sent in real-time to researcher to monitor.  Access and costs NR. | Carers are instructed in the use of the online platform.  Researcher monitors adherence, progression and technical problems.  Carer receives intervention to support CIMT.  Technical support NR | Carer completes 6 modules over 4-6-week period. |
| Chaparro 2018 | Functional capacity.  Includes participants in the sub-acute stroke phase, < 6 months post-stroke.  Excludes participants with severe cognitive or communication deficit.  Excludes participants with disorders limiting gait skills. | **Telephone calls** to encourage physical activity, self-monitoring using SenseWear accelerometer and subjective activity chart, and home visit to review physical activity parameters.  Access and costs NR. | Educational component of intervention but training NR.  Physical activity therapist delivers telephone calls.  Carer and technical support NR. | Telephone call once a week + home visit every 3 weeks, for 6 months. |
| Chau  2019 | Self-efficacy in stroke self-management and HRQoL.  Participants included within 3 months post-stroke to immediately post-discharge.  Excludes participants with severe cognitive or communication deficit.  Carer in dyad must be able to operate the study device kit. | Online platform with educational videos and **video call via Skype** for stroke recovery and self-management tailored based on support **telephone calls**.  Wireless blood pressure monitor recorded in **store and share app**, reviewed by nurse prior to video call.  Each dyad offered **tablet** to access intervention (online video resource, video call and blood pressure app).  Cost effectiveness will be measured. | Nurse meets participants after enrolment to instruct how to use device kit (tablet and blood pressure monitor). Nurses receive 8 x 3-hour training sessions.  Helpline supported by nurse for non-urgent issues (health or technical).  Intervention is also for carers. | Video call of 30-45 minutes, once a month + telephone call once a month, for 6 months.  Weekly blood pressure self-monitoring in app, reviewed remotely by nurse prior to video call. |
| Chen  2018 | Motor function  Include participants <14 days from stroke onset, with unilateral limb movement dysfunction and right-handed.  Excluded participants with dementia and cognitive disorders. | Exercise rehabilitation training and electromyography-triggered neuromuscular stimulation (ETNS) reviewed via **videoconference**.  Clinician platform included **high-quality video-audio system, electronic medical records system and** **remote-control system to guide and review therapy**.  The patient platform includes **high-quality video-audio system, physiological data collection system and muscle electricity biofeedback instrument**.  Network system provides sufficient bandwidth for remote diagnosis, treatment and data collection.  Equipment and examinations provided for free to the participants.  Costs NR. | Protocol mentions improving participant understanding of the trial requirements but specific details of training NR. Researchers receive protocol-specific training.  Specialist physician delivers TR.  Carer to support the exercise and ETNS therapy programme.  Technical support NR. | Weekly review via TR system. Individualised rehab training (OT/PT) for 45 minutes, 5 times a week and ETNS for 20 minutes, 5 times a week, for 3 months. |
| Gauthier 2017 | Upper limb motor function  Include participants >6 months post-stroke with upper limb motor impairment.  Reports aim to include participants usually excluded from trials e.g. cognitive impairment, mobility limitation etc. | **Remotely monitored game-based CIMT** with supplemental **videoconferencing** with therapists plus smartwatch with tri-axial accelerometer for biofeedback.  Compliance -enhancing measures: feedback on adherence, reminder phone-calls, instructional DVD, t-shirt if >90% adherence achieved.  Participants provided with **Kinect gaming system. Cloud-based server** logs play times and skeletal movement data. **Bluejeans videoconference program** for secure communication. Smart watch to record movement + prompts after 10 minutes of inactivity to promote use of the affected upper limb.  Participants are lent **mobile hotspots** during the treatment period.  Costs NR. | Initial consultation involves education on use of technology.  PT remotely monitors use and delivers videoconference consultations.  Carer and technical support NR. | Total dose = 15 h over 3 weeks (10 days of approx. 1.5h, completed in 3 sessions a day with rest); plus 4 in-person consultations; plus 6 video consultations. |
| Guillaumier 2019 | Secondary prevention  HRQoL  Includes participants 3-36 months post-stroke.  Excludes participants with disability at a level that may limit their use of the programme | Online programme accessible using a computer, laptop, tablet or mobile device. Tailored based on participant responses. Text and email prompts for compliance.  Participants required to have access to the internet and device to access the programme.  Cost-effectiveness will be assessed. | Participants receive a letter/email detailing how to access the program.  Clinician support NR.  Carer support will be collected.  Helpline to access project team support. | Participants advised to use programme at least once per week for 12 weeks.  Received fortnightly prompts. |
| Sakakibara 2017 | Secondary prevention / control of risk factors  Includes participants up to one year post-stroke.  Excludes participants with severe cognitive or communication deficit.  Phone access is required. | Lifestyle coaching via **telephone call**.  Telephone required; self-monitoring kit, including Fitbit pedometer, Omron blood pressure monitor, food and activity diaries and health report card, reviewed during coaching calls provided.  Costs NR. | Participant training NR. Lifestyle coach receives protocol-specific training.  Lifestyle coach delivers intervention.  Carer and technical support NR. | 6-month intervention with 2 coaching calls of 30-60 minutes in month 1, then 1 a month thereafter, plus 5 check in calls of 5-10 minutes. |
| Sheehy  2019 | Rehabilitation of sitting and standing balance, gait and upper limb use. Primary outcome of study is the feasibility, acceptance and safety of the virtual reality system at home.  Includes participants discharged from inpatient / outpatient stroke rehabilitation. Time post-stroke/ stroke phase NR.  Excludes cognitive impairment such that they would not be able to learn / use intervention.  Participants require the ability to stand independently ≥2 minutes, have a study partner to assist and sufficient space to use intervention safely. | **Remotely monitored virtual reality training** with t**elephone or email contact** for encouragement, to modify games and address safety/ technical issues. **Asynchronous remote monitoring** of compliance and success, and modification of game difficulty (repetitions, speed, distance and/or accuracy).  Participants provided with **Jintronix Rehabilitation Software and Kinect camera**.  Costs will be measured. | Participant and carer attend 4 training sessions of 45–60 minutes each.  PT installs equipment, delivers training, remotely monitors and contacts weekly via telephone or email.  Carer present throughout sessions.  Logbook for technical issues, addressed by clinician. Participants can contact clinician for support. | Sessions of 30 minutes, 5 times a week, for 6 weeks.  Weekly telephone / email contact with clinician. |
| Sureshkumar 2018 | Function / independence with ADLs  Time post-stroke/ stroke phase NR.  Excludes participants with severe cognitive or communication deficit. | Smartphone enabled education videos with carer support and **telephone support** from clinician to remind and obtain updates on utilisation.  Smartphone application with inbuilt monitoring mechanism to track participant usage.  Participants provided with smartphone loaded with educational videos.  Cost-effectiveness will be assessed. | Stroke survivor and their carer will receive  45–60min of training on accessing videos via the smartphone with competence check.  Clinician NR – likely PT or physician  Carers asked to support participant to access smartphone as needed.  Technical support available. | 6-week intervention period. Asked to use smartphone at their discretion.  Telephone support at least once per week. |
| TR – telerehabilitation  NR – not reported | | OT - occupational therapist  PT - physiotherapist | HR-QOL - health-related quality of life  CIMT - constraint induced movement therapy |  |
