# Supplementary figures and images for: Factors influencing the delivery of telerehabilitation for stroke: A systematic review
Source: PLoS One. 2022 May 11;17(5):e0265828. doi: 10.1371/journal.pone.0265828 (PMC9094559; doi:10.1371/journal.pone.0265828)

Supplementary file 5 – Risk of Bias


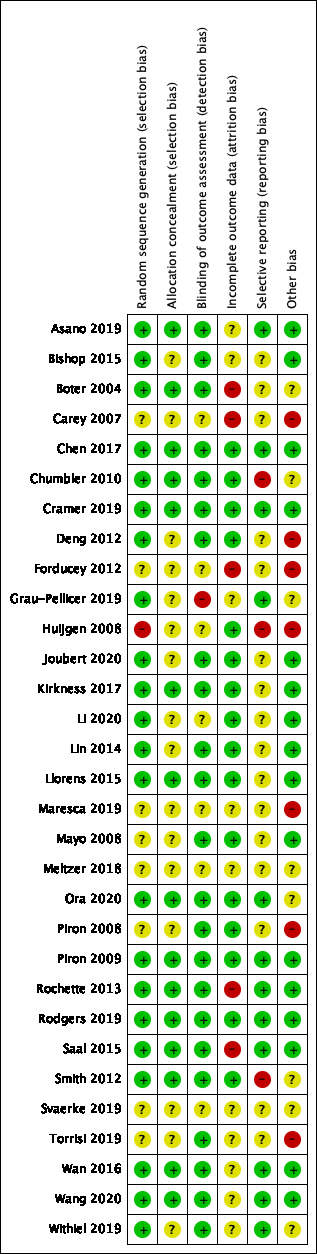


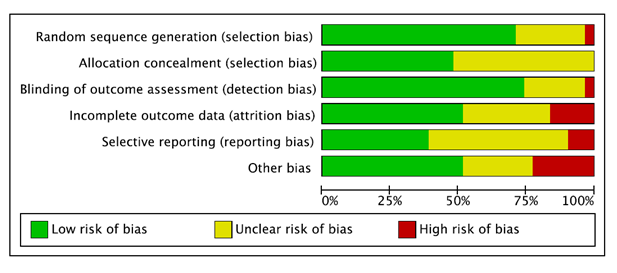

Supplement: S5 File — (DOCX) [file pone.0265828.s005.docx]
