# Supplementary material for: Factors influencing the delivery of telerehabilitation for stroke: A systematic review
Source: PLoS One. 2022 May 11;17(5):e0265828. doi: 10.1371/journal.pone.0265828 (PMC9094559; doi:10.1371/journal.pone.0265828)
Supplement: Attachment — Submitted filename: Plos one response to reviewers 24Sep2021.docx [file pone.0265828.s007.docx]

| Comment | Response | Location |
| --- | --- | --- |
| Introduction Line 68: You cite previous reviews regarding the effectiveness of telerehabilitation for stroke. Please consider including a brief summary sentence regarding this evidence to provide context for the audience regarding this evidence (and its current limitations). | Thank you for this comment, we have amended this sentence in response:  Systematic review evidence for the clinical effectiveness of telerehabilitation for stroke is still emerging, with low or moderate-level evidence that telerehabilitation is a more effective or similarly effective way to provide rehabilitation^10,13-15^. Additionally, the telerehabilitation Interventions and comparisons varied greatly, making it difficult to draw conclusions. | Line 70 |
| Methods Searches were limited to systematic reviews or protocols of systematic reviews. Your research aims looked at exploring facilitators and barriers to use, usability and acceptability. Arguably, data relating to your outcomes of interest would be found in qualitative, or mixed methods studies, please provide justification as to why these were not included in the review, and the limitations that may result from this omission. | Thank you for this comment, we had added this as a limitation:  This review only included RCTs identified from a Cochrane review^13^ and our database searches (RCTs published January 2019 – May 2020); this may have omitted valuable data related to the outcomes of interest of this review available in non-randomised, mixed methods and qualitative studies. Nonetheless, eleven studies included qualitative or quantitative outcomes related to usability and acceptability of telerehabilitation interventions, while remaining information on barriers and facilitators were derived from discussion sections of papers. | Line 492 |
| Lines 112-114: Please provide references for the software used. | We have added the references for the software used. | Line 116 |
| Line 135: Please include a copy of your customised form | We have added our customised form as Supplementary file 2. |  |
| Line 146: Please address the limitations of only 20% of the data extraction being checked | We have revised our limitations section in response to this comment:  For efficiency, data extraction and risk of bias assessment were carried out by a single reviewer with verification (of 20% of data extraction and all risk of bias assessment) by a second reviewer rather than 100% by two independent reviewers, which is recommended to reduce errors where resources permit^83^. | Line 499 |
| Please provide dates of searches carried out so readers can understand the currency of the results. | We have added the date of the database searches. | Line 108 |
| Results: Line 271: Only one study compared two different modes of telerehabilitation delivery and reported that 272 participants reported higher satisfaction and confidence using videoconference compared with telephone call for assessment of their functional status.  Given that telerehabilitation is important in delivering rehabilitation for stroke during the pandemic and beyond, what are the implications for future research and recommendation regarding study designs such as this (comparing different telerehab interventions)? Consider addressing in the discussion | We added the following to our discussion section:  Future research exploring different modes of delivery of telerehabilitation could improve understanding of requirements and optimise telerehabilitation delivery for stroke. | Line 406 |
| Line 286: only one quarter of studies reported adherence – limitations related to conclusions regarding adherence should be addressed | We have slightly amended the wording in the results section to make it more clear that adherence was not consistently reported ( ~~in general~~ -> “where reported” ) and in the discussion we have added an additional limitation that we are unable to draw conclusions due to small numbers of studies reporting on each outcome of interest. | Line 303 |
| Facilitators and barriers to use were reported however these again are taken from a limited number of studies- how were these derived, were these facilitators and barriers included in the results section of the papers, if not what are the limitations of this?  Line 300: In general, telerehabilitation offered participants increased opportunity for therapy29,46 ensure you are not overstating this result as only 2 papers are cited to support this. | We have amended the wording in the results section:  Although facilitators were inconsistently reported, positive feedback for telerehabilitation for stroke included increased opportunity for therapy^29,46^, improved access to, and interaction with, therapists^19,48^ and participants appreciated the telerehabilitation contact provided^20,41^. | Line 312 |
| Risk of bias: The methods section states the studies will be deemed high risk of bias if judged as high risk of bias in any domain. On review of the supplementary material it appears to me that approximately 12 studies have a high risk of bias in at least one domain. There are also many other where the risk is unclear. Could you please clarify your assessment of risk of bias and the impact where the risk is unclear. What impact does this assessment have on the results of your review? | Although we included all the studies in the Supplementary file on risk of bias, in the paper we only report on the eleven additional studies published since the Cochrane review. We have amended the wording to make this more clear (below). Given that effectiveness of interventions was not an outcome of this review, we assessed ROB to explore changes in methodological quality since the Cochrane review rather than to weight our findings. Despite ROB assessment of the included studies, we feel that we could still collect valuable information on intervention delivery for the purpose of this review. The text has been amended as follows, with new text underlined:  Risk of bias was assessed (for completed studies only) by a single reviewer, verified by a second. Where agreement could not be achieved with discussion, a third reviewer completed a consensus assessment. We assessed each study using the Cochrane Risk of Bias tool (V1)^18^ for consistency with Laver^13^, grading on each criterion as having low, high, or unclear risk of bias. A study was judged to be at low risk of bias overall when all domains had a low risk of bias. Conversely, a study was judged to have a high risk of bias when it reported a feature judged as high risk of bias in any domain. Studies with unclear risk of bias in any domain were judged to be at unclear risk of bias. Studies’ risk of bias was not used to weight judgements about findings related to the outcomes of interest of this review. | Line 164 |
| Discussion Technical requirements- given age of the studies what are the implications of changes technology and privacy requirements | We have added the following to our limitations section:  Additionally, given the age of the included studies (18/31 published over three years ago), the present review provides limited practical guidance related to recent advances in technology and changes to data protection requirements. Given its current prominence within health care and health research, future research should ensure clear reporting of privacy and data protection implications. | Line 508 |
| Impact on results and conclusions of high rates of exclusion for participants with cognitive or communication deficits | We have added the following to our discussion:  Those without access to, or who could not use technology were not eligible to participate in over 20% of the studies in the current review, and there were high rates of exclusion for participants with cognitive or communication difficulties. This limits the applicability of our findings to understand how telerehabilitation would be received, in terms of usability and acceptability, by those without access or experience using technology, as well as the clinical transferability for those with residual cognitive or communication difficulties post-stroke. […] Additionally, there is a need for guidelines to support accessibility of interventions for those with cognitive and communication difficulties. | Line 434 |
| Line 397: You make a great point regarding lack of reporting on training. Consider going beyond recommendations for this specifically and looking at overall reporting of these studies which may be strengthened using reporting checklists such as the Tidier checklists which recommend detailed reporting of such things as therapist training. | Thank you for this comment, we have used it to add to this discussion point:  A suggested start for future research could be the use of reporting checklists such as the Tidier checklist^77^, to improve overall reporting of interventions, including clinician training. | Line 425 |
| Line 416: You address impact of exclusion of participants without access to technology, also discussed is people’s hesitancy to participate due to technology – what are the implications for this on results on adherence, usability, satisfaction? | We have added the following sentence ot address this comment:  This limits the applicability of our findings to understand how telerehabilitation would be received, in terms of usability and acceptability, by those without access or experience using technology…. | Line 435 |
| You address need for better economic evaluation, please address quality/rigor of economic evaluations provided in this review. | We have added the following information on the improved reporting of cost effectiveness in the one additional paper including this published since the Cochrane review:  This study^42^ conducted a full cost-effectiveness analysis based on resource utilisation costs and Quality Adjusted Life Years, which was not included in any of the studies previously included in the Cochrane review^12^. | Line 270 |
| Line 445: no safety concerns related to the delivery of telerehabilitation interventions were reported. However, less than a quarter of studies reported adverse events- please ensure you are not overstating this conclusion. | We have amended this sentence to indicate the poor reporting of AEs:  , although adverse events were poorly reported, no safety concerns related to the delivery of telerehabilitation interventions were identified. | Line 468 |
| Line 456 to 458: contains recommendations for telerehabilitation service delivery- please cite evidence supporting this or clarify who is making these recommendations and what this is based on. | We added to this section to improve clarity:  This review identified aspects of good practice and areas for improvement from a number of included studies, to make the following recommendations for service delivery: telerehabilitation should include easy to follow guidelines for use that are tailored to users’ functional abilities and preferences; the opportunity to trial or practice telerehabilitation during a familiarisation period; availability of ongoing technical support; and, options for in-person appointments if required. | Line 480 |
| Limitations: Stroke telerehabilitation is a very broad category and includes different types of interventions aimed at different types of impairments e.g., physical vs cognitive vs communication. Consider acknowledging these differences and the impact this may have on the outcomes you have been addressing in this review. | Thank you for this comment. We have addressed it in our limitations section:  This review included telerehabilitation interventions targeting a range of stroke-related outcomes; given the data available, it is difficult to draw conclusions on any differences in outcomes across different areas of stroke rehabilitation and the implications of these differences. | Line 502 |
| Conclusion: Line 479: The main findings 480 of this review are that stroke telerehabilitation is generally usable and acceptable and can work well 481 with appropriate training and technical infrastructure. However, a significant percentage of studies in the review did not report acceptability and usability- please ensure you are not overstating this conclusion. | We have amended the wording in our conclusion to address this comment:  The main findings of this review are that, where reported, stroke telerehabilitation is generally usable and acceptable and can work well with appropriate training and technical infrastructure. However, given the influence of these outcomes on engagement and sustainability of digital health interventions, it is notable that 65% of studies did not include usability and acceptability outcomes; this should be addressed in future research. | Line 516 |
